# Not All Patients Need Supervised Physical Therapy After Primary Total Knee Arthroplasty: A Systematic Review and Meta-Analysis

**DOI:** 10.7759/cureus.35232

**Published:** 2023-02-20

**Authors:** Yash P Chaudhry, Hunter Hayes, Zachary Wells, Efstratios Papadelis, Harpal S Khanuja, Carl Deirmengian

**Affiliations:** 1 Orthopaedic Surgery, Philadelphia College of Osteopathic Medicine, Philadelphia, USA; 2 Orthopaedic Surgery, Johns Hopkins University School of Medicine, Baltimore, USA; 3 Orthopaedic Surgery, Rothman Orthopaedic Institute, Philadelphia, USA; 4 Orthopaedic Surgery, Thomas Jefferson University, Philadelphia, USA

**Keywords:** musculoskeletal rehabilitation, knee osteoarthritis, home exercise, knee arthroplasty, physical therapy

## Abstract

Although postoperative physical therapy (PT) has long been considered essential to successful total knee arthroplasty (TKA) recovery, recent literature has suggested that unsupervised home exercise regimens may offer similar benefits to formal supervised sessions. We aimed to compare objectively measured physical function and subjective patient-reported outcomes (PROs) between primary TKA patients who received formal supervised physical therapy sessions and those who received unsupervised home exercise regimens after discharge. Six electronic databases were queried to identify randomized controlled trials comparing supervised physical therapy to unsupervised home exercise regimens in primary TKA patients after discharge. Outcomes of interest included change from baseline in objective measures (knee flexion range of motion (ROM), lower extremity strength, and aerobic capacity) and PROs (physical function and quality of life scores). These outcomes were subdivided into short-term (<6 months from surgery; closest data point to three months is used if multiple measurements were made in this time period) and long-term (≥6 months from surgery; closest data point to 12 months is used if multiple measurements were made in this time period) assessments.

A total of 1,884 cases performed in 11 studies were included in this review. There were no significant differences between cohorts with regard to short-term knee flexion ROM (p = 0.7), lower extremity strength (p = 0.6), or patient-reported quality of life (p = 0.5), as well as long-term knee flexion ROM (p = 0.7), patient-reported quality of life (p = 0.2), or patient-reported physical outcome scores (p = 0.3). A small difference in short-term patient-reported physical outcomes was observed in favor of the supervised cohort (standardized mean difference (SMD): 0.3 (95% confidence interval (CI): 0.01, 0.6); I^2 ^= 82%; p = 0.04).

Formal supervised physical therapy regimens do not confer clinically significant benefits over unsupervised home exercise regimens following primary TKA. The routine use of supervised physical therapy after discharge may not be warranted. Further study is needed to determine the subset of patients that may benefit from supervised care.

## Introduction and background

Total knee arthroplasty (TKA) is widely recognized as the gold standard for treating severe osteoarthritis of the knee and achieving satisfaction rates of over 80% for primary procedures [1,2]. As the procedure has become more popular, the patient population has changed. Patients are now younger, have less deformity, and tend to be more active [3,4]. Patients undergoing TKA expect not only decreased pain but also increased quality of life and activity level [5]. To aid in the achievement of these outcomes, rehabilitation and physical therapy (PT) have been viewed as crucial components of the postoperative process [6]. The main emphasis of these PT regimens is to improve quadriceps strength, knee range of motion (ROM), and balance [6]. Despite widespread implementation of intraoperative and postoperative protocols, there remain no formal guidelines for the use of PT or rehabilitation programs following TKA within orthopedic literature. The changing TKA population has called into question the necessity for postoperative supervision of exercise regimens. The aim of this systematic review and meta-analysis was to compare short- and long-term objective physical function markers and subjective patient-reported outcomes (PROs) between primary TKA patients undergoing physical therapy regimens under supervision and those under unsupervised home exercises in the post-discharge period.

## Review

Methods

This systematic review and meta-analysis was subject to Preferred Reporting Items for Systematic Reviews and Meta-Analyses (PRISMA) guidelines [7] and is registered in the PROSPERO International Prospective Register of Systematic Reviews under PROSPERO identifier CRD42021236628. No institutional review board approval was obtained for this study as it did not involve any human subjects.

We included all English language level 1 studies (randomized controlled trials) that directly compared subjective patient-reported outcomes (PROs) and objectively measured physical functional outcomes between patients receiving postoperative PT under the supervision of a trained physical therapist and patients who received home exercise regimens to be performed without supervision following discharge after primary TKA. We limited the time period of intervention from discharge to six months after surgery. Studies were excluded if they did not explicitly state the form of unsupervised home exercise prescribed, which included but was not limited to written exercise regimens given on discharge, video demonstrations of the prescribed exercise regimen, or smartphone application with instructions for exercises to be performed. Studies with preoperative exercise regimens as their primary intervention of interest were also excluded.

To conduct this review, a search was performed on all literature published between the inception of included databases (PubMed, EMBASE, Scopus, Web of Science, Cochrane Library, and ClinicalTrials.gov) and the date of search (12/14/2020). Medical Subject Headings (MeSH) terms were used where applicable to improve the search.

Study Screening

**Figure 1:**
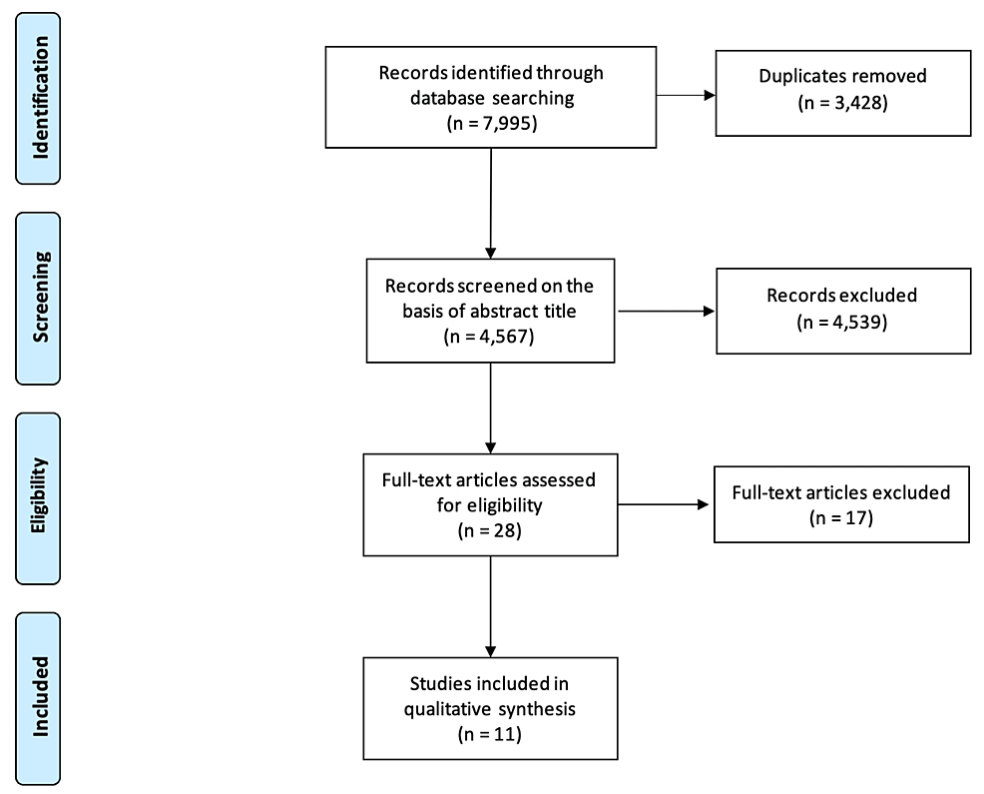
PRISMA flowchart PRISMA: Preferred Reporting Items for Systematic Reviews and Meta-Analyses

A total of 7,995 results were identified (Figure 1), and 3,428 duplicates were subsequently removed. The remaining 4,567 abstracts were screened for inclusion in full-text review independently by two authors (YPC and HH). Twenty-eight articles were identified for full-text review. After the full-text screening, 11 studies remained for inclusion in this review. Any disagreements during the screening process were resolved through discussion involving the screening authors and the senior author (CAD) as needed.

Data Extraction and Quality Appraisal

Extraction of data was performed manually to a master spreadsheet by three authors (YPC, ZW, and HH). The variables of interest included the year of publication, the region in which the study was performed, sample size, patient characteristics (age, gender, and body mass index (BMI)), and study characteristics (inclusion/exclusion criteria, follow-up length, involved therapy/exercise regimens, the time between discharge and initiation of study intervention, the length of intervention, objective physical function indices, and PROs). For objective physical function indices and PROs, change from baseline values was extracted. When not reported, these values were imputed using the methods outlined in the Cochrane Handbook for Systematic Reviews of Interventions [8].

Quality appraisal was performed using the Cochrane Risk of Bias Tool 2.0 [8]. Each outcome was assessed separately and categorized as having low risk (“low”), some concern for risk (“some concern”), or high risk (“high”). Further appraisal and summarization of study quality were performed using the Grading of Recommendations Assessment, Development, and Evaluation (GRADE) system [9].

Statistical Analysis

Outcomes of interest were change from baseline in lower extremity strength, knee flexion range of motion, aerobic capacity, and PROs (physical function and quality of life). These outcomes were split into short-term (between 0 and <6 months; closest time point to three months if multiple time points within this interval were reported) and long-term (between ≥6 and 12 months; closest time point to 12 months if multiple time points within this interval were reported) assessments. Meta-analysis was performed on pooled outcome measures if at least three studies had available data for each measure.

Standardized mean differences (SMDs) were calculated using random-effects models for all outcomes of interest and reported with 95% confidence intervals (CIs). Study heterogeneity was reported using the I2 statistic. P-values below 0.05 were considered statistically significant. All meta-analysis and forest plot generation was performed using Review Manager software version 5.4.1 (The Nordic Cochrane Center, The Cochrane Collaboration, Copenhagen, Denmark). Risk of bias charts were generated using the robvis Risk of Bias Visualization Tool [10].

Results

A total of 1,884 cases in 11 studies were included in this review and meta-analysis (Table 1) [11-21]. All studies were published between 2003 and 2020. All involved starting exercise regimens within eight weeks of surgery, with the length of the program or study interventions ranging from four weeks to 12 months (Table 2). Five studies were conducted in the United Kingdom, two in Australia, and one each in Turkey, the United States of America, Canada, and China. Regarding the timing of interventions, seven studies began their interventions immediately after discharge. Two studies involved interventions starting within two weeks of surgery, while one study began interventions at six weeks and one at eight weeks.

**Table 1:** Study characteristics for primary TKA patients undergoing unsupervised versus supervised exercise regimens following discharge BMI: body mass index, NR: not reported, TKA: total knee arthroplasty, OA: osteoarthritis, PT: physical therapy, RA: rheumatoid arthritis

First author (year)	Country	Number	Mean age (years)	% of women	Mean BMI (kg/m^2^)	Inclusion/exclusion
Artz (2017) [11]	United Kingdom	46	69	52	NR	Inclusion: primary TKA for OA; exclusion: TKA for non-OA reasons, revision TKA, medical limitations precluding exercise, severe neurological disorder, no capacity for informed consent, not fluent in English
Bini (2017) [12]	United Kingdom	28	62	46	NR	Inclusion: unilateral uncomplicated TKA, age ≤ 75, home internet and email access, English fluency, postoperative care performed locally; exclusion: do not meet above criteria
Büker (2014) [13]	Turkey	34	66	91	32	Inclusion: primary TKA; exclusion: NR
Fleischman (2019) [14]	United States of America	290	65	50	31	Inclusion: primary unilateral TKA, age ≥ 18, capacity for informed consent; exclusion: baseline knee flexion ROM < 90 degrees, consideration of disability in the ipsilateral hip or contralateral knee, predetermined discharge to a skilled facility, revision TKA
Hamilton (2020) [15]	United Kingdom	334	68	61	31	Inclusion: primary TKA, OA as indication, baseline OKS ≤ 26; exclusion: unable to comply with PT, no expectation of mobilization postoperatively, revision TKA, PT care pursued non-locally, already received structured PT at six weeks after surgery
Han (2015) [16]	Australia	390	65	54	32	Inclusion: TKA patients aged 45-75 years, able to be discharged home; exclusion: ipsilateral history of UKA/tibial osteotomy, history of hip or knee arthroplasty within previous six months or planned within next 12 months, unable to perform PT exercises at 50%-60% maximum heart rate secondary to medical comorbidities or neurological conditions, RA, unable to attend PT
Ko (2013) [17]	Australia	233	67	63	NR	Inclusion: primarily unilateral or bilateral TKA, planning to perform PT at study institution; exclusion: unable to comprehend study material, unable to participate in interventions secondary to severe medical comorbidity, postoperative limited weight-bearing status, postoperative infection or instability
Kramer (2003) [18]	Canada	160	68	57	32	Inclusion: primary unilateral TKA for OA, ≥90 degrees knee flexion preoperatively, capacity for informed consent, able to follow exercise regimen without supervision; exclusion: RA, disability secondary to a neurological disorder
Mockford (2008) [19]	United Kingdom	143	70	62	NR	Inclusion: primary TKA; exclusion: NR
Rajan (2004) [20]	United Kingdom	120	68	61	NR	Inclusion: primary TKA for monoarticular arthritis, ages 55-90, knee flexion contracture, capable of walking 10 meters without help; exclusion: disability of ipsilateral hip or ankle
Xu (2020) [21]	China	106	68	82	NR	Inclusion: all patients undergoing TKA at participating hospital; exclusion: age < 40 or > 80 years, revision arthroplasty, history of severe systemic disease, lower extremity vascular compromise, or neuromuscular condition, acute trauma or fracture
Total		1,884				

**Table 2:** Study outcomes for primary TKA patients undergoing unsupervised versus supervised exercise regimens following discharge TKA: total knee arthroplasty, U: unsupervised, S: supervised, KOOS: Knee Injury and Osteoarthritis Outcome Score, LEFS: Lower Extremity Function Scale, UCLA: University of California, Los Angeles, VAS: visual analog scale, PT: physical therapy, PS: Physician Function Short Form, VR-12: Veterans Rand 12-Item Health Survey, WOMAC: Western Ontario and McMaster Universities Arthritis Index, ROM: range of motion, SF-36: 36-Item Short-Form Survey, TUG: Timed Up and Go, ADL: activities of daily living, OKS: Oxford Knee Score, 6MWT: 6-Minute Walk Test, SF-12: 12-Item Short-Form Survey, KSS: Knee Society score, NR: not reported

First author (year)	Cohorts	Time from discharge to start	Program length (weeks)	Outcomes assessed
Artz (2017) [11]	U: on discharge, provided a booklet with an exercise regimen, including exercises themselves, guidelines on return to work and other hobbies, precautions and pitfalls to avoid, managing expectations; S: weekly one-hour class for six weeks, given exercises to continue performing in an unsupervised home setting on completion	6 weeks	6 weeks	KOOS, LEFS, UCLA activity, Aberdeen Measures of Impairment, Activities-Specific Balance Confidence scale, Self-Efficacy for Rehab, VAS, Measure Yourself Medical Outcome
Bini (2017) [12]	U: provided iPod with exercise videos and taught how to use the application, patients recorded exercises and uploaded them for PT to review, advanced gradually; S: outpatient PT and rehabilitation clinics	2 weeks	NR	KOOS-PS, VR-12, VAS
Buker (2014) [13]	U: home exercises performed for five days per week, one hour per session; exercises aimed at restoring range of motion, strength in hip and knee muscles; S: 20 sessions of PT program for five days per week; exercises included focus on knee ROM, hip and knee muscle strengthening, transcutaneous electrical nerve stimulation	Upon discharge	4 weeks	WOMAC, VAS, ROM, Beck Depression Scale, SF-36
Fleischman (2019) [14]	U: at discharge, patients received either printed instructions or a link to an interactive web applet for an eight-week unsupervised home exercise program; S: 2-3 weekly sessions with a licensed therapist for 4-8 weeks after surgery	Upon discharge	8 weeks	KOOS, ROM, time back to ADLs/discontinuation of pain medications
Hamilton (2020) [15]	U: six-week unsupervised home exercise program consisting of 18 sessions, exercises focused on improving knee range of motion; S: one-to-one progressive functional PT reviewed and modified weekly for six weeks	8 weeks	6 weeks	OKS, VAS, patient-reported satisfaction, TUG
Han (2015) [16]	U: six-week unsupervised home exercise program involving six exercises performed three times per day, included call with a provider to confirm compliance with exercises and discuss progress; S: postoperative supervised PT regimens recommended by hospital/surgeon, including clinic-based outpatient PT	Upon discharge	6 weeks	WOMAC, ROM, 50-foot walk time
Ko (2013) [17]	U: home-based exercises reviewed with patients prior to discharge; at discharge, patients received physical booklet and instructional DVD exercise program; S: 12-individual (manual therapy, cryotherapy, taping, orthoptics) or group-based (50-minute circuits with group) sessions at the PT department	2 weeks	6 weeks	OKS, WOMAC, VAS, ROM, 6MWT, timed stair ascent/descent, SF-12
Kramer (2003) [18]	U: basic and advanced ROM and strengthening exercises explained in booklets given on discharge, performed three times daily until 12-week follow-up; S: attended outpatient therapy from weeks 2-12 for as many as two one-hour sessions per week	Upon discharge	12 weeks	Knee Society clinical rating, WOMAC, Medical Outcomes Study SF, 6MWT, 30-second stair test, knee flexion ROM
Mockford (2008) [19]	U: home exercise regimen; S: standard outpatient PT regimen	Upon discharge	6 weeks	OKS, Bartlett patellar score, SF-12, ROM
Rajan (2004) [20]	U: home exercise regime given at discharge; S: outpatient physiotherapy regimens (mean of 4-6 sessions performed after discharge)	Upon discharge	NR	ROM
Xu (2020) [21]	U: seven-week home exercise and stretching program performed in 20-minute sessions five days per week; for the remaining 10 months, 2-3 sessions per week; S: seven-week program performed over 24 sessions; for the remaining 10 months, one session per month	Upon discharge	12 months	KSS, ROM, VAS, WOMAC

Meta-Analysis

The summary of all meta-analyses conducted in this study is presented in Table 3. The GRADE assessment is summarized in Appendix A. All aerobic capacity outcomes as well as long-term lower extremity strength outcomes were excluded as there were not enough studies with available data. The risk of bias summary charts are listed in Appendix B.

**Table 3:** Summary of findings SMD: standardized mean difference, CI: confidence interval, ROM: range of motion, LE: lower extremity, QoL: quality of life

Outcome	Supervised participants (number)	Unsupervised participants (number)	SMD (95% CI)	Risk of bias	Certainty of evidence
Knee flexion ROM short term	450	420	-0.1 (-0.8, 0.5)	Low	Low ⨁⨁◯◯
Knee flexion ROM long term	521	492	-0.2 (-1.2, 0.7)	Low	Low ⨁⨁◯◯
LE strength short term	377	281	0.04 (-0.1, 0.2)	Low	Moderate ⨁⨁⨁◯
Self-reported QoL short term	286	182	-0.2 (-0.6, 0.3)	High	Very low ⨁◯◯◯
Self-reported QoL long term	344	241	-0.4 (-1.1, 0.3)	High	Very low ⨁◯◯◯
Self-reported physical outcome short term	778	733	0.3 (0.01, 0.6)	High	Low ⨁⨁◯◯
Self-reported physical outcome long term	634	602	0.1 (-0.1, 0.3)	High	Low ⨁⨁◯◯

**Figure 2:**
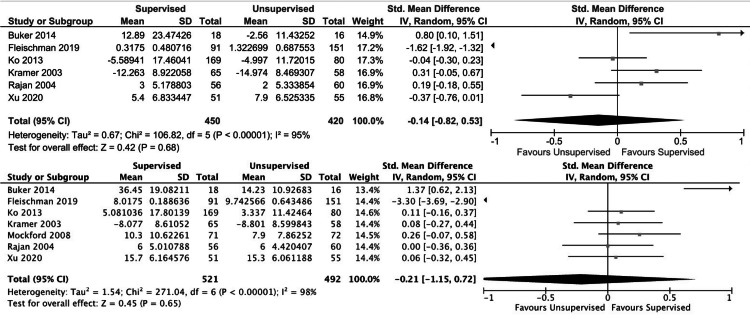
Forest plot comparing short-term (upper plot) and long-term (lower plot) knee flexion ROM in unsupervised versus supervised cohorts SD: standard deviation, IV: weighted mean difference, CI: confidence interval, Chi2: chi-square statistic, P: p-value, df: degrees of freedom, I2: I-square heterogeneity statistic, Z: Z-statistic

**Figure 3:**
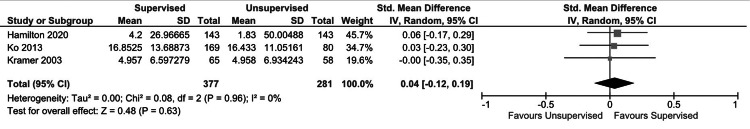
Forest plot comparing short-term lower extremity strength in unsupervised versus supervised cohorts SD: standard deviation, IV: weighted mean difference, CI: confidence interval, Chi2: chi-square statistic, P: p-value, df: degrees of freedom, I2: I-square heterogeneity statistic, Z: Z-statistic

**Figure 4:**
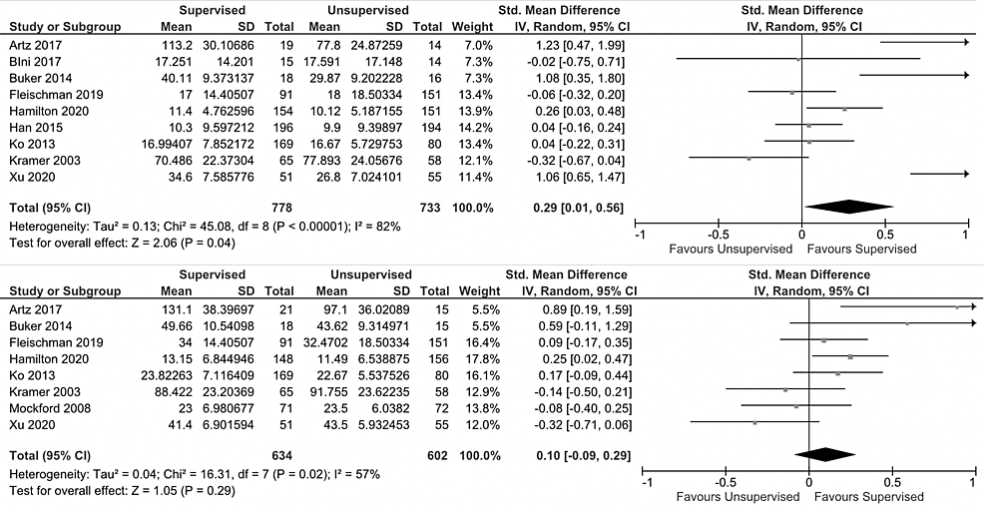
Forest plot comparing short-term (upper plot) and long-term (lower plot) patient-reported physical outcomes in unsupervised versus supervised cohorts SD: standard deviation, IV: weighted mean difference, CI: confidence interval, Chi2: chi-square statistic, P: p-value, df: degrees of freedom, I2: I-square heterogeneity statistic, Z: Z-statistic

**Figure 5:**
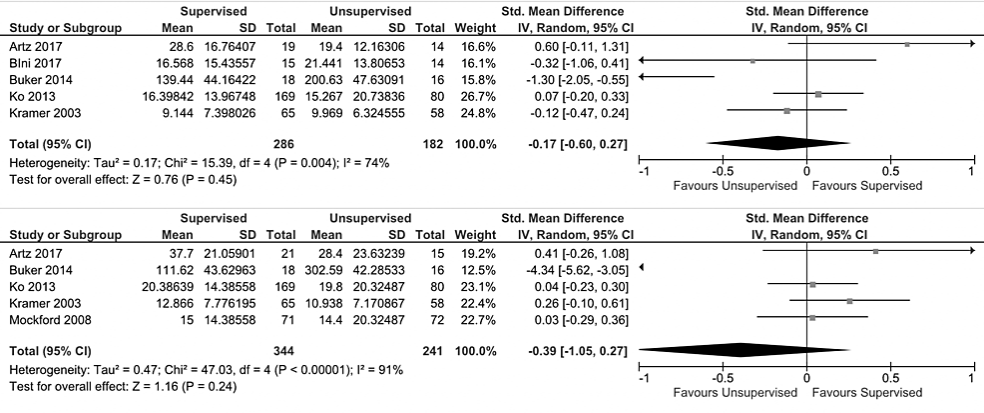
Forest plot comparing short-term (upper plot) and long-term (lower plot) patient-reported quality of life scores in unsupervised versus supervised cohorts SD: standard deviation, IV: weighted mean difference, CI: confidence interval, Chi2: chi-square statistic, P: p-value, df: degrees of freedom, I2: I-square heterogeneity statistic, Z: Z-statistic

For short-term outcomes, no significant differences were found between the unsupervised and supervised cohorts with regard to knee flexion ROM (p = 0.7) (Figure 2, upper plot), lower extremity strength (p = 0.6) (Figure 3), or patient-reported quality of life scores (p = 0.5) (Figure 4, upper plot). There was a small improvement in short-term patient-reported physical outcome scores in the supervised cohort (SMD: 0.3 (95% CI: 0.01, 0.6); I2 = 82%; p = 0.04) (Figure 5, upper plot). No significant differences were found between the unsupervised and supervised cohorts for any of the long-term outcomes: knee flexion ROM (p = 0.7) (Figure 2, lower plot), patient-reported quality of life (p = 0.2) (Figure 4, lower plot), and patient-reported physical outcome scores (p = 0.3) (Figure 5, lower plot).

Discussion

Formal supervised PT and rehabilitation following TKA have historically been viewed as a necessary component for a successful outcome [22]. Our current meta-analysis of the available literature suggests that there is no benefit for supervised PT over unsupervised home exercise regimens for primary TKA patients. No long-term differences between supervised and unsupervised PT were found regarding any of the outcome measures, and no short-term differences were found with regard to lower extremity strength, knee flexion ROM, and self-reported quality of life scores. Our results call into question the traditional notion that supervised PT is of benefit for all patients following primary TKA.

While the use of formal supervised therapy programs after surgery offers several benefits to patients, it does not come without significant drawbacks. One of the largest and most common issues is the lack of convenience in a population of patients for whom travel and access to PT facilities may be a substantial hurdle [11]. Driving or taking public transit to access care can be a painful or burdensome experience for those who do not have family or friends to rely on for support. This was a factor not accounted for in some of the studies included in this review as transportation was actually paid for or provided for subjects in the supervised cohorts as a means to increase compliance [11,17]. In the study conducted by Artz et al., patients either had travel costs subsidized or had taxi transportation arranged for them [11]. The study conducted by Ko et al. also provided taxi or parking vouchers as well as community transit options [17]. While these methods may have increased study compliance, they do not reflect the reality many patients face.

Additionally, this may place a larger burden on patients with a lower socioeconomic status who may be either unable to arrange transportation or afford copays, further adding to the disparity in patient satisfaction between the poor and wealthy after TKA [23,24]. Identifying a successful manner in which to administer unsupervised home exercise regimens could potentially help further close this socioeconomic gap. As access to technology improves over time, this may also fuel discussion for the need to develop web-based programs to help provide access for patients unable to commute to outpatient-based appointments. Additionally, there is a cost reduction benefit associated with unsupervised exercise regimens that were demonstrated by several studies included in this review [13,14,21]. In a retrospective study conducted on 2,971 total joint arthroplasty (TJA) patients in an insurance claims database, Yayac et al. demonstrated that up to 8% of the episode of care costs were directly attributable to formal PT after surgery [25]. Given the recent focus on cost containment in TJA literature due to ballooning healthcare costs, this is another factor to consider in changing the paradigm for routine postoperative care after TKA.

The single outcome that demonstrated a difference in favor of supervised PT was short-term patient-reported physical outcome scores. This benefit did not extend to the long-term assessments, however, and may have been subject to responder bias as patients in the supervised groups were aware of their study assignments. One of the larger studies, a randomized controlled trial conducted by Hamilton et al. involving 334 participants allocated to either an outpatient therapist-led rehabilitation or one-time physiotherapist review with subsequent home-based exercise groups, included only patients thought to perform worse postoperatively (determined by a preoperative Oxford Knee Score (OKS) of <26) [15]. While they demonstrated a slight difference in OKS at 14 weeks, this difference was not statistically significant at 26 or 52 weeks and was well below the minimal clinically important difference (MCID) reported for OKS in previous literature [26].

Additionally, there were no differences found with regard to Timed Up and Go, pain scores, or overall satisfaction with surgery. Due to the focus on patients with preoperative functional deficits, this study likely does not reflect the larger population of TKA patients. This subset of patients warrants further research, but these results suggest that the vehicle for therapy was not important as long as patients performed their assigned exercises. Regarding the other short-term outcomes, no differences were found with regard to lower extremity strength, knee flexion ROM, or patient-reported quality of life. Arthrofibrosis and the possible requirement for manipulation under anesthesia (MUA) remain a chief concern of proponents of supervised PT after surgery [27]; however, studies that examined MUA rates following surgery demonstrated no differences between the supervised and home PT cohorts [14,16-18]. Finally, no long differences were observed in any of the examined outcomes.

Other meta-analyses comparing supervised to unsupervised PT have demonstrated similar results. A meta-analysis conducted by Florez-García et al. in 2017 examined the range of motion and patient-reported physical function in 11 randomized controlled trials and found no significant difference in any assessed outcomes at three, six, or 12 months [28]. Our study differed from this review in several key aspects: we used change from baseline scores as opposed to raw scores and we did not include studies including an accompanying cointervention along with the unsupervised home exercises such as neuromuscular electrical stimulation or continuous passive motion to avoid confounding from those interventions.

Another meta-analysis conducted by Artz et al. in 2015 compared physiotherapy intervention to minimal/no intervention control groups (including home exercise regimens guided by written instruction before discharge) and found improved short-term physical function and pain scores in the physiotherapy group [29]. This study also included an analysis of outpatient versus home-based therapy and demonstrated an improved knee flexion ROM in the home-based group. Again, important differences between this meta-analysis and the current study include the strict inclusion criteria needed to ensure the control (unsupervised group) received some form of exercise intervention. Additionally, in the comparison of home-based versus outpatient therapy, the home-based cohort included supervised home therapy, which may have accounted for the differences in the studies included and subsequent results. Finally, in a meta-analysis conducted by Buhagiar et al. in 2019 of five randomized controlled trials comparing inpatient- or clinic-based therapy to home-based rehabilitation, the home-based group was found to be non-inferior [30]. This study also included supervised home exercise programs in the home-based cohort as well. However, similar to the current study, these meta-analyses reported significant heterogeneity. Despite this, the current review along with prior literature paints an important picture underlining the lack of evidence favoring supervised PT after TKA.

Although this review exclusively considered randomized controlled trials, it is still subject to several key limitations. The inclusion criteria of many of the reviewed studies along with potential selection bias from willing participants may have preselected a healthier population, even accounting for the selection of patients with poor preoperative function in the study by Hamilton et al. [15]. Further study is crucial to determine if certain deconditioned patients or those with preoperative mobility or functional deficits may require supervised skilled oversight during rehabilitation. Additionally, the assessments for physical function scores and quality of life scores are based on patient-reported surveys and may be subject to responder bias as patients are aware of their assignment to either the home exercise or supervised cohorts. Another area of concern is the high heterogeneity reported in our analysis, likely due to the wide range of exercise regimens as well as potential cultural differences and attitudes toward self-driven exercises in different countries.

Additionally, while a cost analysis between the two cohorts would have been useful, this was only reported in two of the included studies [13,21]. Although both demonstrated a substantially higher cost involved in supervised PT as expected, we were unable to perform any data synthesis due to the many factors that could have influenced cost, such as differences in healthcare costs based on country, equipment used in each study, and frequency of therapy visits.

Finally, several studies utilized an intention-to-treat approach, and as such, patients in unsupervised groups may have ended up seeking out supervised therapy such as in the study conducted by Han et al. [16]. Similarly, several patients in the supervised cohort of this study had poor attendance at outpatient visits. However, this is important to consider in actual practice as well, and patient compliance is an issue whether utilizing a supervised or unsupervised postoperative regimen.

The strength of this study lies in the inclusion of randomized controlled trials only. Additionally, we intentionally aimed to make our focus narrower as many studies assessing the efficacy of physical therapy interventions do not describe the care their control groups receive [31].

## Conclusions

This meta-analysis of the currently available literature fails to demonstrate any significant benefit for supervised PT over unsupervised home exercise regimens following primary TKA as a routine practice. Further study is still warranted to determine if select patients such as those with substantial comorbidity burden or preoperative functional deficits may require further skilled therapist intervention. Despite the traditional belief that formal supervised PT is required for successful outcomes, in the setting of a younger patient population and the adoption of early mobilization protocols after surgery, the routine use of supervised PT does not provide any benefit over unsupervised home exercises after primary TKA.

## Figures and Tables

**Figure 6:**
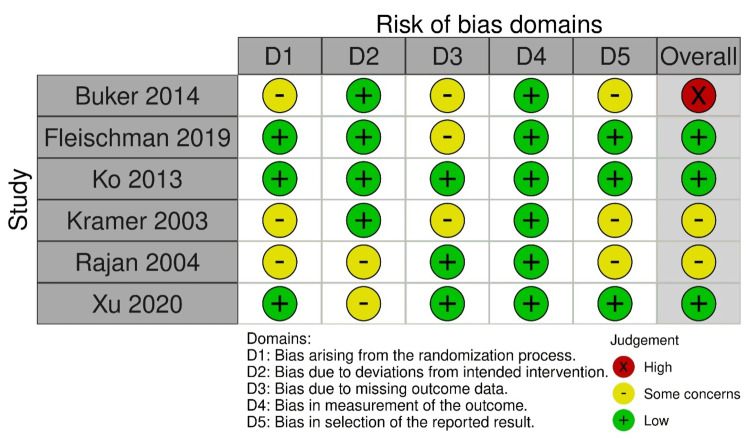
Risk of bias summary chart for short-term change in knee flexion range of motion

**Figure 7:**
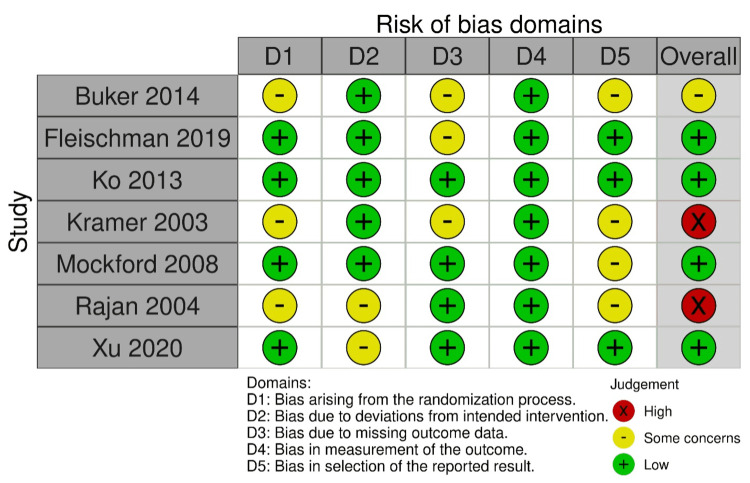
Risk of bias summary chart for long-term change in knee flexion range of motion

**Figure 8:**
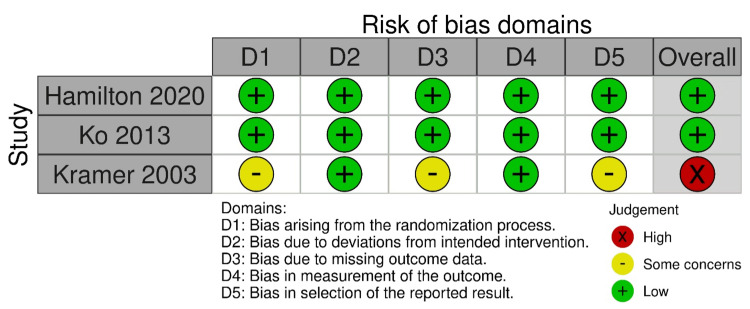
Risk of bias summary chart for short-term change in lower-extremity strength

**Figure 9:**
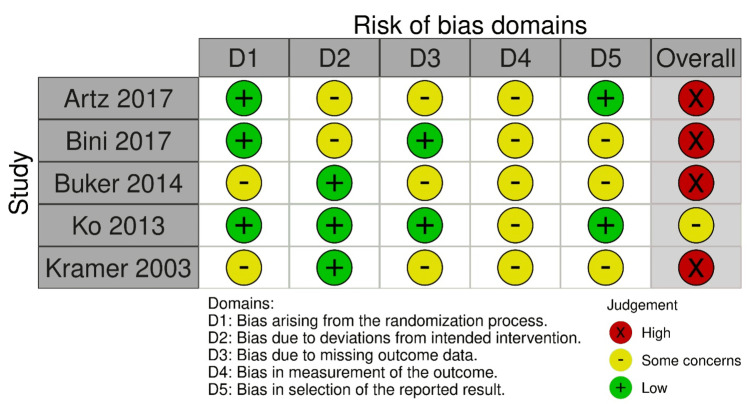
Risk of bias summary chart for short-term change in patient-reported quality of life

**Figure 10:**
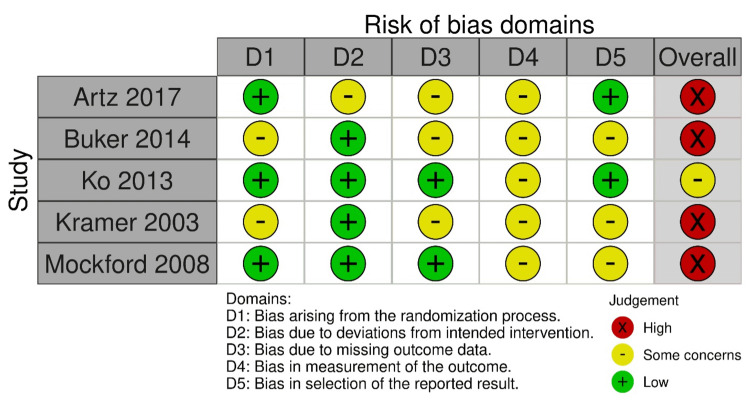
Risk of bias summary chart for long-term change in patient-reported quality of life

**Figure 11:**
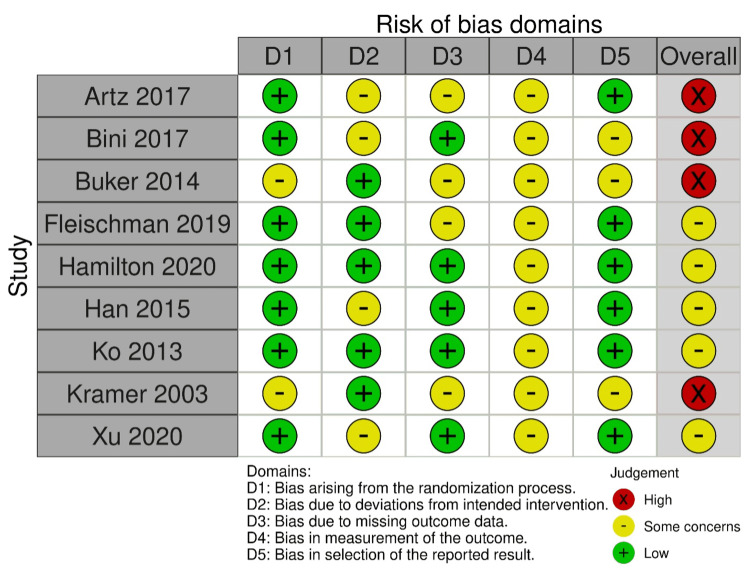
Risk of bias summary chart for short-term change in patient-reported physical function

**Figure 12:**
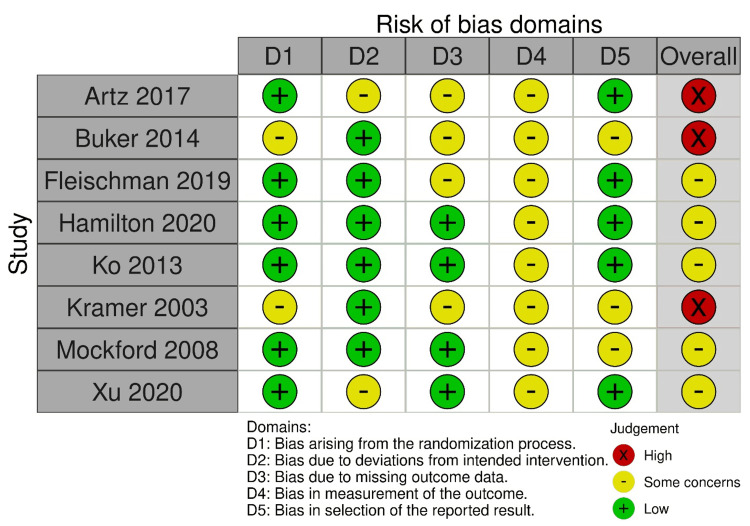
Risk of bias summary chart for short-term change in patient-reported physical function

## Appendices

Appendix A

The GRADE assessment is summarized in Table 4.

**Table 4:** GRADE assessment of meta-analysis results GRADE: Grading of Recommendations Assessment, Development, and Evaluation, ROM: range of motion, I2: I-square heterogeneity statistic, LE: lower extremity, QoL: quality of life

Outcome	Risk of bias	Directness of evidence	Heterogeneity	Precision	Publication bias	Overall quality
Knee flexion ROM short term	No downgrade	No downgrade, no evidence of indirectness	I^2^ = 95%, considerable heterogeneity, downgraded two levels	Low concern for imprecision, no downgrade	No assessment of publication bias conducted	Low
Knee flexion ROM long term	No downgrade	No downgrade, no evidence of indirectness	I^2^ = 98%, considerable heterogeneity, downgraded two levels	Low concern for imprecision, no downgrade	No assessment of publication bias conducted	Low
LE strength short term	No downgrade	No downgrade, no evidence of indirectness	I^2^ = 0%, low heterogeneity, no downgrade	Low concern for imprecision, no downgrade	No assessment of publication bias conducted	Moderate
Self-reported QoL short term	Downgraded by one level, limitation primarily in the measurement of outcome	No downgrade, no evidence of indirectness	I^2^ = 74%, substantial heterogeneity, downgraded one level	Rated down one level, moderate imprecision	No assessment of publication bias conducted	Very low
Self-Reported QoL long term	Downgraded by one level, limitation primarily in the measurement of outcome	No downgrade, no evidence of indirectness	I^2^ = 91%, considerable heterogeneity, downgraded two levels	Low concern for imprecision, no downgrade	No assessment of publication bias conducted	Very low
Self-reported physical outcome short term	Downgraded by one level, limitation primarily in the measurement of outcome	No downgrade, no evidence of indirectness	I^2^ = 82%, substantial heterogeneity, downgraded one level	Low concern for imprecision, no downgrade	No assessment of publication bias conducted	Low
Self-reported physical outcome long term	Downgraded by one level, limitation primarily in the measurement of outcome	No downgrade, no evidence of indirectness	I^2^ = 57%, substantial heterogeneity, downgraded one level	Low concern for imprecision, no downgrade	No assessment of publication bias conducted	Low

Appendix B

The risk of bias summary charts are shown in Figures 6-12.
